# Elevated resting heart rate is associated with several cardiovascular disease risk factors in urban-dwelling black South Africans

**DOI:** 10.1038/s41598-020-61502-4

**Published:** 2020-03-12

**Authors:** N. Peer, C. Lombard, K. Steyn, N. Levitt

**Affiliations:** 1 Non-communicable Diseases Research Unit, South African Medical Research Council (SAMRC), Durban, 4001 South Africa; 2 Department of Medicine, University of Cape Town (UCT), Cape Town, 8001 South Africa; 3 Biostatistics Unit, SAMRC, Tygerberg, 7505 South Africa; 4 Chronic Disease Initiative for Africa, Department of Medicine, UCT, Cape Town, 8001 South Africa

**Keywords:** Type 2 diabetes, Predictive markers, Type 2 diabetes

## Abstract

This study determined the associations of resting heart rate (RHR) with cardiovascular disease risk factors (CVDRF) in 25–74-year-old black South Africans. This cross-sectional study determined CVDRF by administered questionnaires, clinical measurements and biochemical analyses, including oral glucose tolerance tests. Multivariable linear regression models determined the associations of rising RHR with CVDRF. The basic model comprised age, gender, urbanisation, problematic alcohol use, daily cigarette smoking, physical activity and waist circumference. Glucose, blood pressure and cholesterol variables were entered separately and individually in the above model. Among the 1054 participants (382 men and 672 women, mean age 42.8 years), mean RHR was 70.6 beats per minute (bpm) and significantly higher in women (73.6 bpm) compared with men (65.3 bpm). RHR peaked in 45–54-year-old men (69.3 bpm) and 25–34-year-old women (75.3 bpm). Prevalence of RHR < 60 bpm and ≥90 bpm was 24.3% and 6.2%. In the regression model, female gender, problematic alcohol use, decreasing physical activity and increasing waist circumference were significantly associated with rising RHR. All glycaemic variables (diabetes, fasting glucose and 2-hour glucose) and diastolic blood pressure were significantly associated with RHR. The use of RHR in daily primary healthcare settings to identify increased risk for CVDRF should perhaps be encouraged.

## Introduction

Heart rate has evolved from an ordinary clinical index to a relevant cardiovascular risk marker that is associated with poor prognosis. There is a vast body of evidence from epidemiological and clinical studies in diverse sub-groups, including general populations and those with cardiovascular disease (CVD), describing the adverse outcomes associated with elevated resting heart rate (RHR)^1–5^. An elevated RHR has been found to be an independent predictor of all cause, non-cardiovascular and cardiovascular mortality in epidemiological studies^1,2,4–6^. This is independent of the traditional risk factors and other potentially confounding demographic and physiological variables such as age, gender, physical or cardiorespiratory fitness levels, etc^1^. Therefore, elevated RHR is comparable to tobacco smoking, hypertension and dyslipidaemia as a risk factor for CVD^4^.

Although the mechanism whereby elevated RHR wields its harmful effect remains unidentified, several plausible biological mechanisms have been postulated^2^. Among the pathways involved in the link between elevated RHR and mortality, it has been suggested that elevated RHR is indicative of an underlying imbalance in autonomic tone with an increase in RHR likely a reflection of sympathetic overactivity^5,6^. Further, elevated RHR is associated with raised metabolic activity and increased systemic inflammation and is present in the common final pathway of many systemic conditions which involve inflammatory, metabolic, and neurology processes^5^. Consequently, elevated RHR has been found to be associated with incident CVD risk factors such as impaired glucose metabolism and type 2 diabetes mellitus (referred to hereafter as diabetes), obesity, raised blood pressure (BP) and hypertension, dyslipidaemia, smoking, and physical activity levels^2,7–9^.

Nevertheless, to our knowledge, these associations have not been established in populations living in Africa; RHR is an overlooked risk factor for CVD^4^. Moreover, further considerations for the utility of RHR in resource-constrained settings, particularly in Africa, is that it is a simple and cheap clinical sign that is easily obtainable; its measurement is non-invasive and requires no special training or equipment^5,7^. Therefore, the aim of this study was to determine the CVD risk factors associated with elevated RHR in 25–74-year-old black residents in Cape Town, South Africa.

## Methodology

### Study population and sampling procedure

This was a cross-sectional study conducted in a random sample of 25–74-year-old men and women in the predominantly black townships of Langa, Guguletu, Crossroads, Nyanga and Khayelitsha in Cape Town, South Africa. The sampling procedure, which has been described elsewhere in detail^10^, comprised a 3-stage cluster sampling using aerial maps to stratify by area and housing type. Quotas were calculated using the most recent census data and were pre-specified by age and gender categories. It included disproportionate sampling across age groups to ensure at least 50 men and women in each gender category. Participants who were unable to give consent, on tuberculosis treatment or on antiretroviral therapy, bedridden, pregnant or lactating, resident in Cape Town for less than three months or had received cancer treatment within the previous year were excluded from this study. Participants on beta-blockers were excluded from the current analysis.

### Data collection

Data collection was conducted by trained fieldworkers and included administered questionnaires, clinical assessments and biochemical analyses. These were performed in accordance with the relevant guidelines and regulations. Structured questionnaires were administered and collected socio-demographic data, self-reported medical history, physical activity patterns (Global Physical Activity Questionnaire)^11^, tobacco smoking (WHO STEP-wise surveillance questionnaire)^12^ and alcohol use (CAGE questions^13^). Assets that defined wealth including ownership of consumer items (durable goods), access to electricity, and the source of drinking water and toilet facilities, were also recorded.

The clinical examinations included BP, RHR and anthropometric measurements. RHR and BP were measured using an Omron BP monitor with an appropriately sized cuff. Measurements were taken three times at two-minute intervals after participants had been seated for five minutes. The average of the second and third BP and RHR measurements were used in the analyses.

Anthropometric data including height, weight, and waist and hip circumferences were collected using standardised techniques^14^. A calibrated scale and a stadiometer were used to measure weight to the nearest 0.1 kg and height to the nearest 0.1 cm, respectively, with the participants barefoot and in light clothing. A flexible tape measure was used to measure waist and hip circumferences to the nearest 0.1 cm. Waist circumference (WC) was measured approximately 2 cm or two finger spacings above the umbilicus with the tape measure held parallel to the floor. Hip circumference was measured at the maximum posterior protuberance of the buttocks with participants standing upright and their feet together.

Biochemical investigations included oral glucose tolerance tests (OGTT) and fasting lipid estimates with blood samples drawn following an overnight fast of 10 hours. The standard OGTT was thereafter administered and blood samples taken 120 minutes later^15^. Blood samples were kept on ice and transported to the laboratory within six hours to be centrifuged, aliquoted and stored at −80° until the assays were performed.

### Definitions

If two or more of the CAGE set of four questions were answered affirmatively, problematic alcohol use was deemed present^13^. Smoking ≥1 cigarette/day defined participants who smoked daily. Based on the assets that defined wealth, a principal component analysis of the pooled data was used to develop an asset index^16^. Categories of relative wealth were created using tertiles with the lowest tertile representing the poorest participants.

Hypertension was defined as BP ≥ 140/90 mmHg or the use of antihypertensive agents^17^. RHR was categorised as low (<60 beats per minute (bpm)), normal (60–89 bpm) and high (≥90 bpm). Although it may be difficult to identify a RHR at which cardiovascular risk is optimised, these cut-points were selected because risk has been shown to increase continuously with HR > 60 beats/min^1^. Tachycardia or the threshold for raised HR has traditionally been defined as ≥90 or ≥100 beats/min. For example, Diaz and colleagues reported that individuals with low RHR (≤62 bpm) were at lower risk while those with high RHR were at higher risk for cardiovascular mortality/morbidity in a 14.7-year (median) follow-up study of almost 25 000 patients^18^. The CORDIS Study, an eight-year longitudinal study, reported that the risk for cardiovascular death was more than doubled in men with RHR > 90 bpm^19^.

BMI was calculated as weight in kilograms divided by height in metres squared (kg/m^2^) and overweight and obesity defined as BMI ≥ 25 kg/m^2^ ^20^. Central obesity was computed as WC > 94 cm in men and >80 cm in women, WHR as ≥0.9 in men and ≥0.85 in women^21^ and WHtR as >0.5^22^.

Diabetes was diagnosed according to the 1998 WHO definition of fasting plasma glucose ≥7.0 mmol/l and/or 2-hour post glucose load ≥11.1 mmol/l^15^ or participants with known diabetes. Dyslipidaemia was defined as follows^23^. total cholesterol (TC) > 5 mmol/l, triglycerides >1.5 mmol/l, high-density lipoprotein cholesterol (HDL-C) < 1.2 mmol/l and low-density lipoprotein cholesterol (LDL-C) > 3.0 mmol/l calculated using the Friedewald equation^24^ or taking anti-lipid agents.

### Statistical analysis

Data analyses were conducted using STATA 15.0. Descriptive statistics, including prevalence, were calculated using the weights based on the sample design and adjusted for the realised sample. Univariate analyses (socio-demographic and CVD risk characteristics) are presented as mean values and standard deviations (SD) for continuous data, and as percentages for categorical data.

Multivariable linear regression analyses determined the independent associations of the sociodemographic variables, lifestyle behaviours and adiposity with RHR in a main effects model. Of the adiposity measures, WC was selected for the basic model because of its well-established link with cardiometabolic diseases compared with the other adiposity variables. Thereafter, each cardiometabolic variable was modelled independently.

Ethical approval was obtained from the University of Cape Town’s Research and Ethics Committee. All participants signed informed consent.

## Results

After excluding 17 participants who did not fulfil the inclusion/exclusion criteria, the realised sample consisted of 1099 participants. Of the individuals invited to participate, 84% of men and 87% of women accepted. There were 187 selected individuals who the study team were unable to contact i.e. the non-responders; 79 (42%) of whom were men. For this analysis, participants who were on beta-blockers were additionally excluded (n = 45), leaving 1054 participants (382 men and 672 women).

**Figure 1:**
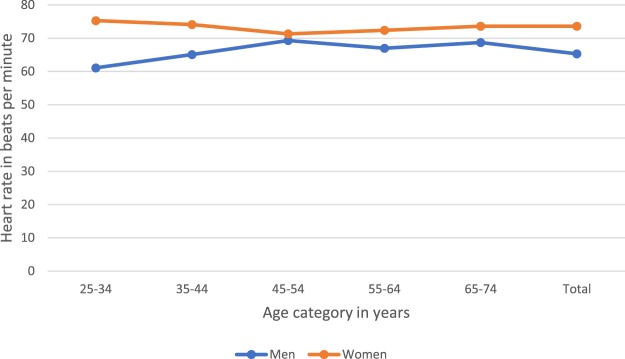
Distribution of mean resting heart rate by age and gender categories.

The mean age of study participants was 42.8 years (SD ± 12.7) **(**Table 1**)** and similar in men (43.0 ± 12.6) and women (42.6 ± 12.7) (p = 0.652). However, while the mean RHR was 70.6 bpm (SD ± 12.4) overall, it was significantly higher in women (73.6 ± 11.3) compared with men (65.3 ± 12.4) (p < 0.001) **(**Fig. 1**)**; the gap was particularly wide in 25–34-year-olds at 75.3 bpm in women and 61.1 bpm in men. There was no clear pattern by age category in men and women except for those younger than 55 years of age **(**Fig. 1**)**. In men, RHR increased with older age, peaking in 45–54-year-old men at 69.3 bpm. In contrast, RHR peaked in 25–34-year-old women and was lowest in 45–54-year-old women (71.3 bpm). RHR was significantly different by age categories in men (p < 0.001) and women (p < 0.008).

**Table 1:** Socio-demographic and lifestyle characteristics presented by resting heart rate category.

Heart rate (beats per minute)	Total	<60	60–89	≥90	p-value
Number (%)	1054	200 (24.3)	779 (69.5)	75 (6.2)	
***Socio-demographic characteristics***					
Age in years, mean ± SD	42.8 ± 12.7	40.0 ± 11.6	43.5 ± 12.8	42.3 ± 12.9	**0.010**
Gender, %:					**<0.001**
Male	48.0	81.1	38.5	24.5	
Female	52.0	18.9	61.5	75.5	
% of life spent in urban area (years), mean ± SD	61.4 ± 32.7	61.8 ± 30.5	61.4 ± 33.2	60.9 ± 33.7	0.817
Education: ≤7 years of schooling, %	33.7	35.2	33.4	31.4	0.860
Employment Status, %:					0.272
Employed	23.1	26.1	22.8	14.7	
Unemployed	61.7	61.4	60.9	72.7	
Other*	15.2	12.5	16.4	12.6	
Housing type: informal shack, %	51.9	55.6	50.4	54.4	0.461
Lowest asset index/wealth tertile, %	33.5	39.6	30.7	39.8	**0.031**
***Lifestyle behaviours***					
Problematic alcohol use: CAGE ≥ 2, %	33.7	41.5	31.5	27.6	**0.036**
Smoke: ≥1 cigarette/day, %	28.2	48.9	22.0	16.0	**<0.001**
Physical activity (minutes/week), mean ± SD	1051.7 ± 940.8	1211.6 ± 1098.0	1034.7 ± 901.2	801.5 ± 823.3	**0.001**

Mean ± SD are reported for the study sample and not adjusted for the population; *Other: comprised pensioners, homemakers, students and those receiving disability grants; significant p-values are in bold.

About a quarter (24.3%) of participants had RHR < 60 bpm, and 6.2% had RHR ≥ 90 bpm **(**Table 1**)**. Most participants with RHR < 60 bpm were men (81.1%) while 75.5% of those with RHR ≥ 90 bpm were women. RHR was not significantly associated with the other socio-demographic characteristics.

With regards to lifestyle behaviours, the prevalence of problematic alcohol use (p = 0.036) and smoking ≥1 cigarette/day (p < 0.001) were significantly and inversely related to higher RHR **(**Table 1**)**. The amount of moderate to vigorous physical activity per week also decreased significantly with greater RHR (p < 0.001).

Mean adiposity levels as determined by BMI, WC and WHtR increased significantly with higher RHR category (all p < 0.001) **(**Table 2**)**. Fasting and 2-hour glucose levels increased significantly with rising RHR (both p < 0.001). Diastolic (p = 0.001), but not systolic (p = 0.085), BP increased significantly with greater RHR. Of the lipid variables, triglycerides increased significantly with RHR (0.009).

**Table 2:** Cardiometabolic characteristics presented by resting heart rate category.

Heart rate (beats per minute)	Total	<60	60–89	≥90	p-value
Number (%)	1054	200 (24.3)	779 (69.5)	75 (6.2)	
**Mean** ± **SD**					
Adiposity					
Body mass index (kg/m^2^)	29.6 ± 8.4	25.5 ± 6.5	30.5 ± 8.4	31.3 ± 9.2	**<0.001**
Waist circumference (cm)	92.9 ± 15.6	85.5 ± 13.3	94.4 ± 15.4	96.7 ± 17.4	**<0.001**
Waist-to-hip ratio	0.86 ± 0.1	0.86 ± 0.1	0.86 ± 0.1	0.88 ± 0.1	0.312
Waist-to-height ratio	0.57 ± 0.1	0.51 ± 0.1	0.58 ± 0.1	0.60 ± 0.1	**<0.001**
Glucose (mmol/l)					
Fasting	5.4 ± 2.5	4.9 ± 1.5	5.4 ± 2.6	6.0 ± 3.6	**<0.001**
2-hour	6.9 ± 4.2	5.8 ± 3.2	7.0 ± 4.2	8.3 ± 5.8	**<0.001**
Blood pressure (mmHg)					
Systolic	125.2 ± 22.9	128.4 ± 21.5	124.7 ± 22.9	124.8 ± 26.2	0.085
Diastolic	81.8 ± 13.2	79.6 ± 12.7	82.0 ± 13.0	85.8 ± 15.9	**0.001**
Lipids (mmol/l)					
Total cholesterol	4.4 ± 1.1	4.3 ± 1.0	4.4 ± 1.1	4.5 ± 1.7	0.148
High-density lipoprotein cholesterol (HDL-C)	1.2 ± 0.5	1.2 ± 0.3	1.2 ± 0.5	1.1 ± 0.5	0.209
Triglycerides	1.1 ± 0.9	1.0 ± 0.8	1.1 ± 0.7	1.4 ± 2.2	**0.009**
Low-density lipoprotein cholesterol (LDL-C)	3.0 ± 1.0	2.9 ± 0.9	3.0 ± 0.9	3.1 ± 1.2	0.051
**Prevalence, %**					
Adiposity					
Body mass index ≥25 kg/m^2^	56.4	31.9	63.8	69.8	**<0.001**
Waist circumference: men >94 cm, women >80 cm	53.8	25.9	61.9	72.9	**<0.001**
Waist-to-hip ratio: men ≥0.9, women ≥0.85	41.6	24.9	46.3	54.0	**<0.001**
Waist-to-height ratio >0.5	61.8	34.5	70.3	72.6	**<0.001**
Diabetes	11.5	4.8	13.1	19.3	**0.002**
Hypertension	35.8	28.4	38.0	39.8	**0.035**
Dyslipidaemia					
Total cholesterol >5 mmol/l	23.8	17.2	25.9	25.0	**0.049**
HDL-C < 1.2 mmol/l	61.0	58.6	61.3	68.1	0.434
Triglycerides >1.5 mmol/l	15.6	10.1	17.7	13.3	**0.027**
LDL-C > 3 mmol/l	41.7	35.4	43.9	42.7	0.135

Mean ± SD are reported for the study sample and not adjusted for the population; Hypertension: BP ≥ 140/90 mmHg or on hypertension treatment; Diabetes: fasting glucose ≥7.0 mmol/l, 2-hr glucose ≥11.1 mmol/l or known diabetes; significant p-values are in bold.

The prevalence of overweight and obesity by all parameters examined increased significantly by RHR (all p < 0.001) **(**Table 2**)**. Diabetes (p = 0.002) and hypertension (p = 0.035) increased significantly with higher RHR category. Hypercholesterolaemia (p = 0.049) and triglyceridaemia (0.027) differed significantly by RHR category peaking in participants with RHR 60–89 bpm.

In the basic multivariable linear regression model, female gender (coefficient: 7.412, 95% CI: 5.289–9.533, p < 0.001), problematic alcohol use (2.239, 0.355–4.123, p = 0.020), physical activity (−0.001, −0.002–0.0002, p = 0.012) and increasing WC (0.011, 0.005–0.017, p < 0.001) were significantly associated with rising RHR **(**Table 3**)**. When the cardiometabolic variables were individually entered in this basic model, there was no change in the direction or significance of the original variables. All dysglycaemia variables i.e. diabetes (4.519, 1.683–7.356, p = 0.002) and higher fasting (0.628, 0.281–0.975, p < 0.001) and 2-hour (0.526, 0.284–0.768, p < 0.001) glucose levels were significantly related to higher RHR in separate models. Rising diastolic BP (0.111, 0.046–0.177, p = 0.001), but not hypertension or systolic BP, was associated with higher RHR when entered separately in the basic model. None of the dyslipidaemia variables were significantly related to RHR in individual models.

**Table 3:** Multivariable linear regression model for the associations with increasing resting heart rate.

	Coefficient	95% Confidence Interval		p-value
		Lower limit	Upper limit	
Increasing age	0.028	−0.049	0.104	0.476
Gender: female	7.412	5.289	9.533	**<0.001**
Greater proportion of life spent in urban area	−0.005	−0.030	0.020	0.685
Problematic alcohol use: CAGE ≥ 2	2.239	0.355	4.123	**0.020**
Smoke: ≥1 cigarette/day	−1.771	−4.204	0.662	0.153
Increasing physical activity levels (minutes/week)	−0.001	−0.002	−0.0002	**0.012**
Increasing waist circumference (cm)	0.011	0.005	0.017	**<0.001**
Diabetes	4.519	1.683	7.356	**0.002**
Increasing fasting glucose (mmol/l)	0.628	0.281	0.975	**<0.001**
Increasing 2-hour glucose (mmol/l)	0.526	0.284	0.768	**<0.001**
Hypertension	0.989	−0.736	2.714	0.260
Increasing systolic blood pressure (mm Hg)	−0.028	−0.069	0.012	0.165
Increasing diastolic blood pressure (mm Hg)	0.111	0.046	0.177	**0.001**
Total cholesterol >5 mmol/l	1.647	−0.286	3.580	0.094
High-density lipoprotein cholesterol <1.2 mmol/l	−0.696	−2.467	1.076	0.439
Triglycerides >1.5 mmol/l	1.442	−0.607	3.490	0.167
Low-density lipoprotein cholesterol >3 mmol/l	0.182	−1.424	1.787	0.824

When the cardiometabolic variables were individually entered in the basic model, there was no change in the direction or significance of the other variables; significant p-values are in bold.

## Discussion

This study, to our knowledge, is among the first to describe the relationship between RHR and CVD risk factors in Africa. We found significant associations between rising RHR and female gender, problematic alcohol use, decreasing physical activity and increasing WC. Further, rising RHR was significantly associated with dysglycaemia and increasing diastolic BP.

The differences in mean RHR by gender and the trends by age category require some consideration. The higher RHR in women compared with men is in keeping with other studies and likely due to the central role played by the autonomic nervous system in the regulation of cardiovascular homeostasis^25^. It’s been suggested that women have higher parasympathetic and lower sympathetic influences on RHR than men; therefore, they have a higher set point for RHR. This gender difference in autonomic nervous system activity may be related to dominant sex hormone levels. Further, in this study, the greater physical activity levels in men compared with women (1165.1 vs. 987.2 mean minutes/week, p = 0.003, data not shown) and their subsequent better cardiorespiratory fitness may have contributed to the lower mean RHR in men compared with women.

The highest RHR was in the youngest women, which was unexpected and may highlight the complexities of heart rate dynamics. For example, these are women in the child-bearing age who may be prone to menorrhagia, which may lead to anaemia and subsequent elevated RHR. Further research is required to confirm and explain this association. All dysglycaemia variables i.e. diabetes and increasing fasting- and 2-hour-glucose levels were significant related to rising RHR in this study. There are several biological mechanisms to expound the relation between RHR and diabetes^9^. Among the suggested pathways for the association is a possible imbalance between parasympathetic and sympathetic activity that favours an elevated RHR. Further, greater sympathetic tone increases insulin resistance; therefore, impaired autonomic nervous activity may play an intermediary role in the relationship between RHR and diabetes. Another mechanism may be the activation of the sympathetic nervous system in the presence of metabolic syndrome, abdominal obesity and insulin resistance; elevated RHR would then be considered a consequence rather than a cause of the metabolic alterations. Autonomic dysfunction is associated with increased mortality risk in diabetes probably because autonomic imbalances increase the susceptibility to cardiac arrythmias^26^. Therefore, individuals with diabetes may not only be at increased risk for poorer outcomes because of their diabetes but perhaps also because of their significantly higher RHRs.

The association of rising RHR with dysglycaemia in this study accords with the findings of a systematic review that included 10 cohort studies and >5628 cases and 119,915 participants^9^. However, none of these cohorts were from Africa; therefore, while the current study is cross-sectional in design, its findings are directly relevant to local populations. More research is required to unravel the causal mechanisms^9^ and to demonstrate the role of RHR in incident diabetes in South African populations. If a clear link is demonstrated, RHR may potentially play a clinical role in resource constrained developing world settings, seeing that it is simple, cheap and non-invasive to determine. Improving RHR may perhaps be incorporated into intervention programmes aimed at decreasing the risk of diabetes^9^.

Interestingly, raised triglycerides were associated with elevated RHR in the univariate analyses but not in the multivariable regression model. This may be a manifestation of the link between raised triglycerides and dysglycaemia/diabetes^27^.

The absence of a significant association between RHR and hypertension or systolic BP is surprising considering that longitudinal studies have shown a link between raised RHR and the development of hypertension^3,8^. However, the aetiology of hypertension is multi-factorial and complex with black African populations more predisposed to the condition. Other factors may perhaps play a greater role in their susceptibility to hypertension. For example, sodium retention and volume expansion are key contributors to the development of hypertension in black Africans^28^. Therefore, the latter may have contributed to increased circulating volume and lower RHR in this sample of predominantly younger participants (mean age 42.8 years), particularly in men. Further research is required to clarify this relationship in African populations.

Nevertheless, while no link was found between hypertension or SBP and rising RHR, DBP was significantly associated with elevated RHR in this study. A cross-sectional study in Japan reported significant associations of both SBP and DBP with elevated RHR in age and gender adjusted models^29^, which partially accords with our findings. The hypothesised mechanisms for causal relationships between higher RHR and hypertension or rising BP include arterial stiffness, endothelial dysfunction, sympathetic overactivity, and the activation of inflammatory pathways^3^.

Despite the association of elevated RHR as a long-term predictor of cardiovascular outcomes in high-risk individuals with hypertension, decreasing the RHR has not been found to be always valuable^30^. One of the reasons may be the unfavourable effects of beta-blockers on lipid profile and insulin resistance. Therefore, in hypertensive individuals without cardiac disease, lowering RHR may not convey any additional advantage^30^.

The significant association of rising RHR with increasing WC in this study is of concern. In view of the high prevalence of obesity in South Africa, particularly in women^31^, the association of elevated RHR with shorter life expectancies^2^ is an added impetus for concerted action to address the obesity epidemic. Of note is that this association was independent of physical activity levels. Our findings were in keeping with longitudinal studies that have reported significant links between RHR and adiposity as determined by BMI^2,29^.

The decreasing prevalence of cigarette smoking and problematic alcohol use by increasing category of RHR in the univariate analyses is likely related to the lower proportion of men with RHR ≥ 60 bpm compared with women, and that men were more likely to smoke cigarettes and drink compared with their counterparts. Among those that smoked cigarettes daily in this study, 85% were men and 15% were women (data not shown). Of the problem drinkers, 72% were men and 28% were women (data not shown).

Nevertheless, in the multivariable linear regression model, problem drinking which reflects chronic alcoholic misuse, was significantly associated with rising RHR. Problematic alcohol use may favour cardiac stimulation and subsequently an elevated RHR through greater sympathetic and/or decreased parasympathetic activity^32^. Studies have shown that acute alcohol intake disrupts vagal activity.

In the multivariable linear regression model, cigarette smoking was not significantly linked to rising RHR, similar to the findings from a cross-sectional study conducted in Japan^29^. Nevertheless, an association between RHR and smoking has been reported in other studies^2,6^. The suspected mechanism is postulated to be via the negative effect that smoking has on arterial stiffness over time and which may impact the RHR^6^.

A few limitations of this study include the cross-sectional study design; while we have demonstrated several associations between RHR and CVD risk factors, we are unable to infer causality. Further, although three readings were taken, RHR and BP were measured only at one instead of multiple time points. The lower sample realisation in men necessitated higher sampling weights and a loss of precision. The self-reported histories of tobacco and alcohol use may have resulted in an underestimation of these risky behaviours on account of their perceived social unacceptability, particularly for smoking in women and for alcohol in general. However, the CAGE questionnaire used to assess problem drinking is an indirect measure that has been found to be reliable in this setting. These limitations notwithstanding, this study provides valuable insights on the associations of RHR with CVD risk factors in an African setting.

## Conclusion

RHR was found to be significantly associated with several CVD risk factors in this study. Considering that elevated RHR is indicative of poor outcomes and that it is a simple, cheap and quick clinical index to determine, its daily use in primary healthcare settings to identify increased risk for CVD risk factors should perhaps be reviewed and encouraged.
